# Creatine as a Promising Component of Paternal Preconception Diet

**DOI:** 10.3390/nu14030586

**Published:** 2022-01-28

**Authors:** Sergej M. Ostojic, Tonje Holte Stea, Dagrun Engeset

**Affiliations:** 1 Department of Nutrition and Public Health, University of Agder, P.O. Box 422, 4604 Kristiansand, Norway; dagrun.engeset@uia.no; 2 FSPE Applied Bioenergetics Lab, University of Novi Sad, 21000 Novi Sad, Serbia; 3 Department of Health and Nursing Sciences, University of Agder, P.O. Box 422, 4604 Kristiansand, Norway; tonje.h.stea@uia.no; 4 Department of Child and Adolescence Mental Health, Sørlandet Hospital, 4604 Kristiansand, Norway

**Keywords:** fertility, energy metabolism, creatine, nutrition, creatine kinase

## Abstract

Male fertility has been declining globally over the past several decades, advancing from a personal issue to a public health problem. Beyond any doubt, a reduction in fertility (often characterized by low sperm count or motility) can severely threaten reproductive health and lifecourse framework in a long-term fashion. Aside from uncovering the currently unknown etiology of modern-day male infertility, the scientific and medical community faces a double burden: finding an efficient biomarker of impaired fertility and exploring any intervention that can act to enhance fertility. A plethora of nutritional compounds have been recognized as possible modulators of semen quality, and specific dietary patterns and nutrients appear to be accompanied by a lower risk of male infertility. Creatine, a conditionally essential nutrient, has caught attention as a male fertility-promoting candidate due to its role in sperm energy metabolism. This mini-review describes the creatine-related bioenergetics of spermatozoa, explores a connection between creatine levels and sperm quality in men, and critically examines available evidence for interventional studies with creatine to affect sperm viability.

## 1. Background

According to the Global Burden of Disease study, global fertility rates have been dropping steadily whereas life expectancy has been increasing over the past 20 years [1]. Although the factors behind this worldwide decline in fertility rate remain largely unclear [2], a drop in semen quality represents a significant public health issue in terms of reproductive and lifecycle health [3]. Besides other factors, poor nutrition has been recognized as a possible disruptor of semen quality, and the Western-style diet appears to be accompanied by a higher risk of male infertility [4,5]. Healthy dietary patterns, on the other hand, correlate well with better sperm milieu, and a smaller risk of abnormalities in parameters such as sperm count, sperm concentration, and motility [6]. Whole-diet interventions, such as the Mediterranean diet [7] or plant-based diet [8], as well as individual nutrients, including zinc [9], selenium [10], and omega-3 fatty acids [11], are put forward as nutritional models that could support male fertility. Recognizing other dietary interventions able to enhance sperm quality and support paternal preconception capacity remains of the utmost interest for both the research community and the general public. Moreover, as male infertility is a complex biological and social phenomenon, identification of valid biomarkers for infertility diagnosis has been requested [12]. Creatine, a conditionally essential nutrient and a popular dietary supplement [13], drives attention as another male fertility-promoting candidate due to its role in sperm energy metabolism. This mini-review describes creatine-related bioenergetics of spermatozoa, explores a connection between creatine levels and sperm quality in men, and critically examines available evidence for interventional studies with creatine to affect sperm viability.

## 2. Semen: An Energy-Demanding Fluid

**Figure 1:**
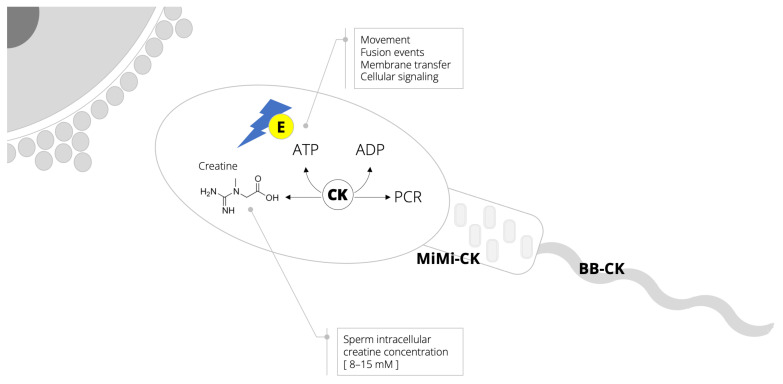
Creatine–phosphocreatine (PCR) shuttle and high-phospate energy (E) production and utilization in spermatozoa. Abbreviations: ATP, adenosine triphosphate; ADP, adenosine diphosphate; CK, creatine kinase; MiMi-CK, mitochondrial CK isoform confined to the midpiece region rich in mitochondria; BB-CK, tail-specific CK isoform localized within the sperm tail but not in the head portion.

As a male reproducing fluid that predominantly contains spermatozoa (along with various organic and inorganic compounds), semen exhibits exceptional resilience to withstand environmental stress and promote the survival of cells prior to and during conception. This is mostly due to the fact that spermatozoa can effectively sustain high and fluctuating energy requirements [14]. A continuous supply of high-energy phosphates in sperm requires a significant contribution of the creatine–phosphocreatine shuttle (Figure 1), a critical metabolic pathway in cellular bioenergetics [15]. Total creatine content of spermatozoa (8–15 mM) and seminal plasma (~4 mM) [16,17] are comparable to levels found in other energy-demanding cells, such as skeletal and cardiac myocytes, and photoreceptor cells of the retina [18]. Among different functions, creatine in spermatozoa is involved in the phosphocreatine shuttle, thereby shuttling energy (adenosine triphosphate, ATP) from the mitochondria to the contractile machinery to fuel movement but also fertilization, cellular transport, and other metabolic reactions. Creatine kinase is also indispensable for sperm function because it catalyzes the regeneration of energy from the shuttle [19]. Sperm high-energy production is compartmentalized, with two distinct creatine kinase isoenzymes found in the sperm tail and midpiece region rich in mitochondria [17]. Interestingly, the inactivation of creatine kinase can impair the pattern of sperm motility [20]. Having this in mind, the evaluation of creatine–phosphocreatine shuttle biomarkers is often used as a tool to monitor sperm health [16,21] (Box 1).

Box 1 **Sperm quality vs. fertility.**

Previous studies have used both sperm quality and fertility interchangeably, and therefore the terms are used in the same way in this mini-review. However, the reader should be aware that the use of sperm quality as a marker for male fertility is highly debated. Sperm quality mainly concerns the number of spermatozoa and their viability, motility, and morphology. Although these are all factors that lower the chance of conception, they are rarely the only cause of infertility. Other factors, such as the man’s age or his partner’s age, may be contributing factors to the success of conception. Thus, poor sperm quality does not necessarily predict infertility [22].

## 3. Biomarkers of Creatine Metabolism and Sperm Quality

A possible link between semen creatine metabolism and sperm quality in humans has been debated for almost 60 years. In a preliminary communication published in *The Lancet* back in 1963, Lehmann and Griffiths [23] suggested that extremely high concentrations of creatine kinase found in seminal fluid (385–14,000 IU) might be used in tracking azoospermia. This seminal article was followed by a handful of reports describing creatine kinase levels in fertility studies [24,25,26], with nearly all suggesting a relative value of the enzyme activity as an indicator of spermatogenesis. Arguably the first study about the seminal concentration of creatine and sperm viability was reported by Srivastava and co-workers [27]. The authors demonstrated that creatine levels tended to be higher in normal males than in infertile counterparts, suggesting the remarkable importance of creatine for spermatozoa quality. Huszar et al. [28] confirmed qualitative metabolic differences among the sperm of oligospermic and normospermic men, with a highly significant inverse correlation between sperm creatine kinase activities and sperm concentrations. The inverse relationship between creatine kinase levels and sperm concentration and morphology was found in sub-fertile men, implying that elevated creatine kinase levels may reflect biochemically immature spermatozoa [29]. Another study found that the mean creatine kinase levels in the severely oligospermic group were 18-fold higher than that in the moderate and mild groups, with creatine kinase higher in all three infertile groups compared with the donor group [30]. Interestingly, the concentration of two isoforms of creatine kinase (CK-B and CK-M) in normozoospermic and two groups of oligozoospermic patients were significantly different, with CK-M levels correlated negatively with sperm concentration and sperm motility, but correlated positively with the pathologic sperm form [31]. A recent trial reported that low semen creatine levels are associated with reduced sperm motility, while high creatine kinase activity is associated with poor sperm quality [16]. Interestingly, various lifestyle factors can impair sperm bioenergetics and creatine kinase activity, as well as sperm motility, including smoking [32] and environmental exposure to pesticides [33]. The above findings suggest a relationship between compromised creatine–phosphocreatine metabolism and low sperm count/activity; recovering normal creatine turnover in spermatozoa thus might help males with poor quality sperm.

## 4. Exogenous Creatine and Sperm Viability

Utilizing exogenous creatine to improve sperm quality has been investigated in a handful of in vitro and animal studies so far. The addition of creatine phosphate to the insemination media enhances the fertilizing capacity of sperm (both motility and velocity) during in vitro fertilization [34]. Creatine also enhances sperm capacitation by increasing adenosine triphosphate levels when added to in vitro fertilization medium [35]. Indeed, successful fertilization was achieved with as few as five sperm in the creatine group, and the number of fertilized oocytes was significantly higher than in the control group without creatine. Creatine induced and sustained zig-zag sperm motility and improved the fertilization ability of boar sperm under hypoxic conditions when added to in vitro fertilization medium [36]. A dietary administration of creatine precursor, guanidinoacetic acid, was associated with the improvement in semen concentration, total sperm number, and sperm forward motility (also sperm penetration and fertility rate) in broiler breeder roosters [37]. An interesting cross-sectional study in 778 young, healthy men taking protein supplements (of those, 44% men reported using creatine) found that semen concentration and total sperm count tended to be higher in current users than in never users (42 vs. 36 million/mL, and 108 vs. 90 million, respectively) [38]. Although preliminary, the above studies provided the first evidence about the potential effects of exogenous creatine in tackling sperm quality; this strongly justifies further interventional and mechanistic studies with dietary creatine in a real-life context of male (in) fertility. Specifically, it remains unknown how dietary creatine is delivered to spermatozoa, and whether infertility may compromise creatine uptake. Preclinical studies demonstrate an expression of testis-specific creatine transporter (CT2) [39,40], with its role in creatine uptake (sequestration of creatine from the plasma and/or creatine transport within the reproductive tract) remains to be clarified.

## 5. Paternal Preconception Diet with Creatine: The Future Steps

Building male fertility through the diet might be a simple, convenient, and straightforward strategy to tackle this fundamental element of reproductive health. Working towards this goal requires many studies employing various dietary routines and nutrients, and creatine could be the next promising agent in the pipeline. In terms of lifecycle nutrition, dietary creatine has been confirmed as a particularly important compound in female reproduction, pregnancy, and newborn health (for a detailed review, see Ref. [41]), and for the normal growth of children and adolescents [42]. Dietary intake of creatine in very young children (0–24 months) is roughly three times larger than that of the adult population [43], implying its critical role in optimal brain development for this sensitive population. A summary of experimental studies suggests a protective role of maternal peri-conception diet complemented with creatine to improve fetal and neonatal morbidity and reduce mortality in high-risk human pregnancy [44]. Whether a dietary intake of creatine of father at the conception, and even before, influences fertility biomarkers (along with the health of their future children) currently remains unknown. A first step in addressing this question might require re-evaluating fertility cohort data by exploring possible associations between the intake of creatine-containing foods (e.g., fish, meat, milk) and fertility biomarkers in men, followed by well-designed long-term randomized controlled trials with dietary creatine.

## 6. Conclusions

As energy-demanding cells, spermatozoa can suffer from deficient creatine metabolism, with poor sperm count and motility are often associated with low creatine levels. A provision of supplemental creatine appears to positively affect sperm quality in pilot trials, suggesting a potential for using creatine to attenuate sub-fertility. This is accompanied by favorable safety profile of creatine supplementation reported in the scientific and medical literature so far, with short and long-term supplementation (up to 30 g/day for 5 years) is safe and well-tolerated in healthy individuals and in a number of patient populations ranging from infants to the elderly [45]. However, to become recognized as a functional component of a paternal preconception diet, dietary creatine has a long journey ahead that should start with exploring its intervention in men with low sperm concentration.

## Data Availability

Not applicable.
